# More than meets the eye: mutation of the *white* gene in *Drosophila* has broad phenotypic and transcriptomic effects

**DOI:** 10.1093/genetics/iyaf097

**Published:** 2025-05-17

**Authors:** April Rickle, Krittika Sudhakar, Alix Booms, Ellen Stirtz, Adelheid Lempradl

**Affiliations:** Department of Metabolism and Nutritional Programming, Van Andel Institute, 333 Bostwick Avenue, Grand Rapids, MI 49503, USA; Department of Metabolism and Nutritional Programming, Van Andel Institute, 333 Bostwick Avenue, Grand Rapids, MI 49503, USA; Department of Metabolism and Nutritional Programming, Van Andel Institute, 333 Bostwick Avenue, Grand Rapids, MI 49503, USA; Department of Metabolism and Nutritional Programming, Van Andel Institute, 333 Bostwick Avenue, Grand Rapids, MI 49503, USA; Department of Metabolism and Nutritional Programming, Van Andel Institute, 333 Bostwick Avenue, Grand Rapids, MI 49503, USA

**Keywords:** *Drosophila*, *white* gene, genetic background, backcrossing, behavioral phenotyping, transcriptomics

## Abstract

The *white* gene, one of the most widely used genetic markers in *Drosophila* research, serves as a standard background mutation for transgene insertions and genetic manipulations. While its primary function involves eye pigmentation, mutations in *white* have been associated with diverse phenotypic effects, including those related to metabolism, behavior, and stress responses. However, many of the published studies do not account for differences in genetic background, raising concerns about the interpretation of experimental results. To address this, we generated fly lines through 10 generations of backcrossing that are highly genetically similar except at the *white* locus, minimizing background variation. Given the likely metabolic consequences of *white* gene deletion and its role in neurotransmitter production, we focused on behavioral, metabolic, and fitness-related traits and performed transcriptomic analysis on adult fly heads. Our findings both confirm and refine previous observations, revealing that some reported effects of *white* mutation are robust while others likely reflect underlying genetic background differences. These results emphasize the necessity of genetic background control in *Drosophila* research and warrant caution when using *white* mutants as a baseline for comparative studies.

## Introduction

First described in 1910 by Thomas Hunt Morgan (Morgan 1910), the *white* (*w*) gene encodes an ABC class transporter involved in the intracellular transport of eye pigment precursors (tryptophan, guanine, and kynurenine) and other small molecules (GMP, guanine, amine, riboflavin, xanthine, zinc, and pyruvate) (Sullivan and Sullivan 1975; Sullivan *et al*. 1979, 1980; Mackenzie *et al*. 1999; Borycz *et al*. 2008; Ferreiro *et al*. 2018; Myers *et al*. 2021; Sasaki *et al*. 2021). Its disruption prevents pigment biosynthesis, resulting in white eyes. Due to resource availability and ease of selection, functional *white* (w^+^) has been extensively used as a marker for transgene insertions into *white* mutant (w^−^) backgrounds (Green 2010). Although often considered a “neutral” mutation, numerous studies report phenotypic effects across a wide range of biological processes, including behavior, neurodegeneration, metabolism, lifespan, immunity, and oxidative stress (Geer and Green 1962; Zhang and Odenwald 1995; Hing and Carlson 1996; Anaka *et al*. 2008; Borycz *et al*. 2008; Elias *et al*. 2008; Ambegaokar and Jackson 2010; Simon *et al*. 2012; Krstic *et al*. 2013; Chan *et al*. 2014; Zalucki *et al*. 2015; Navrotskaya *et al*. 2016; Xiao and Robertson 2016; Qiu *et al*. 2017; Xiao *et al*. 2017; Ferreiro *et al*. 2018; Myers *et al*. 2021; Sasaki *et al*. 2021). Some of these phenotypes can be attributed to retinal degradation due to pigmentation loss and inability to filter excess light. This deficit has long been associated with abnormalities in behaviors requiring sight (Borycz *et al*. 2008; Simon *et al*. 2012; Xiao *et al*. 2017; Ferreiro *et al*. 2018). However, *white*'s role in pigmentation cannot explain differing phenotypes such as locomotor degeneration, recovery from anoxia and anesthetics, activity, olfactory learning, cholesterol homeostasis, and lifespan (Kalmus 1943; Zhang and Odenwald 1995; Campbell and Nash 2001; Anaka *et al*. 2008; Dubruille and Emery 2008; Elias *et al*. 2008; Oxenkrug 2010; Zalucki *et al*. 2015; Xiao and Robertson 2016; Qiu *et al*. 2017; Xiao *et al*. 2017; Ferreiro *et al*. 2018; Myers *et al*. 2021). Unfortunately, the use of diverse w^−^ fly lines (isoCJ1, w^1118^, w^1^, w^a^, w^a4^, and w^+^) and w^+^ lines (w^e^; Canton-S, Oregon-R, Vallecas, g^1^, and ry^50^) in such publications makes comparisons difficult (Kalmus 1943; Zhang and Odenwald 1995; Campbell and Nash 2001; Anaka *et al*. 2008; Dubruille and Emery 2008; Elias *et al*. 2008; Oxenkrug 2010; Zalucki *et al*. 2015; Xiao and Robertson 2016; Qiu *et al*. 2017; Xiao *et al*. 2017; Ferreiro *et al*. 2018; Myers *et al*. 2021). Additionally, due to their short lifespan, small population sizes, and repeated bottleneck events, fly lines maintained separately for many generations are bound to diverge genetically (Dobzhansky and Spassky 1962; Briscoe *et al*. 1992; García-Dorado *et al*. 2007). This genetic drift and accumulation of genetic variations over time has been observed in laboratory stocks dating back to 1962 (Dobzhansky and Spassky 1962). Most of the above studies do not control for genetic background, with only a minority of studies addressing this critical variable (Simon *et al*. 2012; Chan *et al*. 2014 ; Xiao *et al*. 2017; Myers *et al*. 2021; Sasaki *et al*. 2021).

A systematic comparison of w^+^ and w^−^ flies is essential to clarify the broader physiological consequences of this widely used genetic marker. To minimize background differences, we generated fly lines through 10 generations of backcrossing that are highly genetically similar except at the *white* locus. We then assessed the impact of *white* mutation across multiple biological domains. Behavioral traits were evaluated using locomotor, activity, and social spacing assays; metabolic traits through measurements of starvation resistance, triglyceride levels, and oxidative stress; and fitness traits via egg laying, hatching success, lifespan, and immune response. Finally, we performed transcriptomic analysis of adult fly heads to characterize gene expression changes. This integrated approach allowed us to disentangle the specific contributions of *white* mutation from confounding background effects and to reevaluate previous findings under controlled genetic conditions.

## Materials and methods

For reagents, see Supplementary Table 14.

### Fly husbandry and lines used

Flies were maintained at 25°C at 60% humidity on a 12-h dark/light schedule. Ageing flies were kept 25/vial and flipped weekly onto fresh fly food M without Tegosept using all organic ingredients from LabExpress, Ann Arbor, MI, USA. Fly lines used were w^1118^ line (RRID:BDSC_3605) and Harwich line (obtained from Daniel Hartl at Harvard). FlyBase was used throughout the course of this study (Öztürk-Çolak *et al*. 2024).

### Backcrossing

We backcrossed a wild-type *white* allele from a Harwich female into the w^1118^ background over 10 generations. In each generation, red-eyed virgin female offspring were mated to w^1118^ males (Supplementary Fig. 1). Offspring from the 10th generation included red-eyed and white-eyed males and females in a 1:1:1:1 ratio. These flies were used in all phenotyping assays, except those involving embryos or larvae (e.g. hatching, immune, and oxidative stress), where eye color could not be used to distinguish genotypes. For those assays, we generated homozygous lines. White-eyed males and females from the 10th cross were mated to establish a homozygous w^−^ line (cross 11W), while red-eyed males and females were mated to establish a homozygous w^+^ line (cross 11R). To reduce the chance of including heterozygous flies, red-eyed pairs from cross 11R were single-pair mated (cross 12R). Offspring from vials that produced only red-eyed flies were retained, and lines were monitored for any emergence of white-eyed flies to confirm homozygosity.

### Activity assay

The activity assay was conducted as previously reported (Pfeiffenberger *et al*. 2010). Briefly, single flies were placed into glass capillary tubes with food at one end and a plug at the other. Tubes were placed in the TriKinetics DAM2 monitor, and flies were allowed to acclimate for roughly 10 h before the start of the assay period. The flies' movement was measured in 1-min intervals for the next 48 h. Each time a fly crossed the light beam from the DAM2 halfway across the tube being counted as 1 “activity count.” The *Drosophila* sleep and circadian analysis MATLAB program (SCAMP) was used for analysis to generate various measures of sleep and activity levels from the raw activity counts. Principal component analysis (PCA) of the measurements for all different SCAMP output parameters was performed in R (Vecsey *et al*. 2024).

### RING assay

To assess locomotor function, we employed an automated variant of the original rapid iterative negative geotaxis (RING) assay (Warner Gargano *et al*. 2005). Twenty-four hours prior to testing, 10 flies per replicate were transferred into fresh vials containing standard food. At the time of the assay, flies were transferred into empty plastic vials without CO_2_ and placed into the Locomotor Activity Monitor (LAM25, TriKinetics), which was mounted atop a standard multitube vortex (Talboys) with the tubes in the upright position. The assay consisted of 20 consecutive cycles, each involving 3 s of shaking at the fourth intensity setting every 30 s (total duration: 10 min). This setting reliably knocked all flies to the bottom of the vial without causing harm. After each shake, flies were allowed to climb, and flies crossing the light beam at the midpoint were recorded at 5, 10, 15, 20, and 25 s postshake. The average number of climbs per fly was calculated for the 25-s interval. To prevent cumulative effects of repeated mechanical stress, each weekly time point used flies from the same cohort that had not previously undergone RING testing.

### Social spacing assay

Social spacing was measured using a modified version of previously described methods (Simon *et al*. 2012). All testing was conducted at the same time of day, between ZT5 and ZT9. Flies were briefly anesthetized with CO_2_ and separated into groups of 20 before being placed in a vertical triangular arena (height = 13″, base = 13″). Once all flies had recovered from anesthesia, they were gently tapped to the bottom of the arena to ensure a uniform starting position. Flies were allowed to settle into their preferred social spacing for 20 min, and an image was captured for analysis. The position of each fly was identified using ImageJ, and the distance to its nearest neighbor (NND) was determined.

### Triglyceride assay

Triglyceride content was measured via colorimetry in 8-day-old males and mated females as previously described (Tennessen *et al*. 2014) with minor adjustments. Ten flies were used for each assay, and 5 replicates per condition and sex were performed. The total weight of flies per replicate was recorded before flash freezing in liquid nitrogen. Tubes were placed on ice, and approximately 0.2 g of Lysing Matrix D beads (MP Biomedicals) and 400-µL PBST (0.05% Tween) were added. Samples were homogenized using a FastPrep-24 Machine at 6 m/s for 20 s and then briefly spun down in a microcentrifuge before incubating at 70°C for 10 min. Samples were homogenized again using the same settings and then spun down at 10,000 rpm for 5 min at room temperature. Forty microliters of supernatant from each sample was transferred to a 96-well plate, with 2 technical replicates per sample. Two hundred microliters of triglyceride working reagent (Sigma) was added to each replicate, and 2 wells of 240-µL blank reagent were added to the plate. The plate was sealed and incubated in a 37°C water bath for 10 min. The plates were then spun at 4,000 rpm for 5 min at room temperature, and 100 µL of each replicate was transferred to a new plate to be read on a Synergy Neo2 plate reader at 540 nm.

### Starvation resistance assay

Eight-day-old adult male and mated female flies were separated under mild CO_2_ anesthesia and transferred into vials containing 3 mL of solidified agar media. The assay was set up with 5 replicates per sex and eye color and 20 flies per replicate. The number of dead flies was recorded every 2 h from 6 Am to 10 Pm daily until all flies had died. The value for each replicate represents the time for all 20 flies in the replicate to die.

### Lipid peroxidation

Quantification of lipid peroxidation using BODIPY 581/591 C11 (Invitrogen) was performed as previously described (Kilwein *et al*. 2023). Briefly, embryos or newly hatched L1 larvae were collected and washed in 1× TSS (0.4% *w*/*v* NaCl, 0.03% *v*/*v* Triton-X) before being separated into replicates of 10 individuals each in 15 µL of 1× TSS. Sixty-three-microliter 2× TSS, 60-µL BODIPY solution (1 µL of 5 µm BODIPY 581/591 C11 lipid peroxidation sensor in DMSO per 1-mL ddH_2_O), and approximately 10–20 Lysing Matrix D beads (MP Biomedicals) were added to each sample. Samples were homogenized using a FastPrep-24 Machine at 6 m/s for 30 s and then spun down in a microcentrifuge at 6,000 rpm for 1 min before incubating 30 min in the dark at room temperature. Blank samples consisted of 63-µL 2× TSS, 60-µL BODIPY solution, and 15-µL 1× TSS. One hundred microliters of each sample was transferred to a 384-well plate and read at 485 and 561 nm on a Synergy Neo2 plate reader. Values were calculated by subtracting the blank values from the sample values and then dividing the 485-nm reading by the 591-nm reading.

### Egg laying assay

Egg laying was assessed for virgin and mated females across 3 successive age groups: age 11 (days 10–12), age 21 (days 20–22), and age 31 (days 30–32). The assay used 20 females per replicate with 6 replicates per group over 3 days, with each day counted separately. For nonvirgin females, flies were mated 24 h before the assay. Plates were changed and eggs were counted every 12 h.

### Hatch and embryo survival assay

Eggs from 10- to 14-day-old, mated females were collected between 3 and 4 Pm and separated into replicates of 100 eggs in PBS. From 5 Am to 8 Pm the next day, all hatched larvae were removed and counted hourly. Larvae that hatched before 5 Am or after 8 Pm were excluded from the average hatching time calculation due to inability to accurately confirm time of hatching, but these larvae were included in the hourly hatching and embryo survival data.

### Longevity assay

Newly hatched flies from cross 10 were collected under mild CO_2_ anesthesia and separated into vials based on sex and eye color, with 10 flies per vial and 10 replicates per sex/eye color combination. Vials were checked daily for fly deaths and flipped onto new food every Tuesday and Friday. The assay continued until all flies had died. Lifespan was recorded as the number of days posteclosion. Each replicate value represents the average lifespan of the 10 flies in that replicate.

### Immune assay

Innate immune response was assessed using previously defined methods (Dudzic *et al*. 2015). Briefly, L3 wandering larvae were collected using a 4 M sucrose solution (150-g sucrose/L) added to each vial to allow larvae to float to the top. Male and female larvae were then separated, and each individual was poked with a 0.25-mm tungsten needle in the cranial third region. The larvae were subsequently transferred to a 96-well plate containing fresh 4 M sucrose solution, and after 15 min, larvae exhibiting melanization (brown dot) were counted. Due to high daily variability in the assay, data were normalized to day and sex matched median red eye values.

### RNA isolation

RNA was isolated from 1-week-old unmated males and females. For each sample, 10 heads were removed under mild CO_2_ anesthesia and flash frozen in liquid nitrogen. Five hundred microlteres of Trizol (Invitrogen) and approximately 0.2 g of Lysing Matrix D beads (MP Biomedicals) were added to each sample, and samples were homogenized using a FastPrep-24 Machine at 6 m/s for 30 s 3 times with a 5-min rest on ice in between homogenizations. Samples were vortexed for 15 s and incubated at room temperature for 5 min before adding 100 µL of chloroform, vortexing another 15 s, and incubating at room temperature for 5 min. Samples were centrifuged at 12,000 × *g* for 15 min at 4°C. Two hundred microliters of the upper aqueous layer was transferred to a new tube, where 1 µL of GlycoBlue and 225-µL ice-cold isopropanol were added. Samples were incubated 10 min at room temperature before centrifuging at 12,000 × *g* for 15 min at 4°C. The supernatant was removed, and the pellet was washed twice with 1-mL 75% ethanol and then spun at 7,500 × *g* for 5 min at 4°C to remove the 75% ethanol. Samples were allowed to dry for 10 min before rehydrating with 15-µL nuclease-free water and incubating at 60°C for 5 min.

### RNA sequencing

Libraries were prepared using 500-ng RNA and the Kapa mRNA Hyperprep Kit (Roche) according to the manufacturer's protocol. Samples were sequenced using an Element AVITI to a depth of 30 M reads and a run length of 2 × 75 bp.

### Differential expression, gene set enrichment, and overrepresentation analysis

Low-quality read ends were trimmed, followed by adapter sequence removal using TrimGalore v0.6.1 (Krueger 2019). The—quality and—length parameters were set to 20. Trimmed reads were then aligned to the r6.60 version of the *Drosophila* genome (dos Santos *et al.* 2015) using STAR v2.5.4b (Dobin and Gingeras 2015) with default parameters. ReadsPerGene.out.tab files were imported into R v4.4.1 (R Core Team 2021) to obtain a counts table. DESeq2 v1.44.0 (Love *et al*. 2014) was then run to detect differentially expressed (DE) genes (Supplementary File 1). Gene set enrichment was performed in R using gene set enrichment analysis (GSEA) 4.3.3 (Subramanian *et al*. 2007), gene set size filters (min = 10; max = 500), weighted enrichment statistics, and Signal2Noise gene ranking. .gmt files for enrichment analysis were generated using code presented in “reference code to generate gmt file.” The results of the enrichment analysis were displayed in a network plot created using Cytoscape 3.10.1 (Shannon *et al.* 2003), plotted are pathways with *q* < 0.1 and overlap similarity coefficient > 0.1 (Fig. 4c). Annotation clusters were identified using AutoAnnotate (Kucera *et al*. 2016). Results of the GSEA and cluster analysis can be found in Supplementary Tables 2–5. Counts and statistical analyses for up- and downregulated (adjusted *P* < 0.05) gene overlaps between males and females were done in R using the GeneOverlap package v1.40.0 (Shen 2024) (Fig. 4b; Supplementary File 2). DE genes with an adjusted *P*-value cutoff of <0.05 were used to perform the overrepresentation analysis in WebGestalt (2024 release) (Elizarraras *et al*. 2024) (Fig. 4d–g; Supplementary Files 3 and 4).

### Statistics

All statistics were calculated in GraphPad Prism unless stated otherwise. In the RING, activity, social spacing, body weight, triglyceride content, starvation resistance, egg laying, median lifespan, immune reactivity, and hatching by hour assays, we used a full effects model 2-way ANOVA with Šídák's multiple comparisons to determine significance. The lipid peroxidation data did not pass D’Agostino and Pearson testing for normality, so a Mann–Whitney test was used to determine significance. For the survival/longevity assay, we used a log-rank test to determine significance. For the hatching time and embryo survival assays, we used an unpaired *t*-test. To test for significance of differences in expression of *white*, we used a Brown–Forsythe and Welch ANOVA with a Dunnett multiple comparisons test. The activity assay PCA was done in R using the prcomp function.

## Results

To establish a working model, we performed ten generations of backcrossing to insert a wild-type copy of *white* from a Harwich line into the commonly used w^1118^ line (RRID: BDSC_3605), whose loss of function stems from a partial deletion (Hazelrigg *et al*. 1984) of *w*. Heterozygote females from generation 10 were then crossed with w^1118^ males to yield 1:1:1:1 w^+^ males, w^+^ females, w^−^ males, and w^−^ females (Supplementary Fig. 1). The progeny was then used to determine the effects of loss of *white*. Age and sex matched flies are used for all experiments and comparisons. For all figures, data for red-eyed flies are represented in red and data for white-eyed flies are represented in gray.

### Behavioral phenotyping reveals differences in activity patterns and climbing ability

**Fig. 1.**
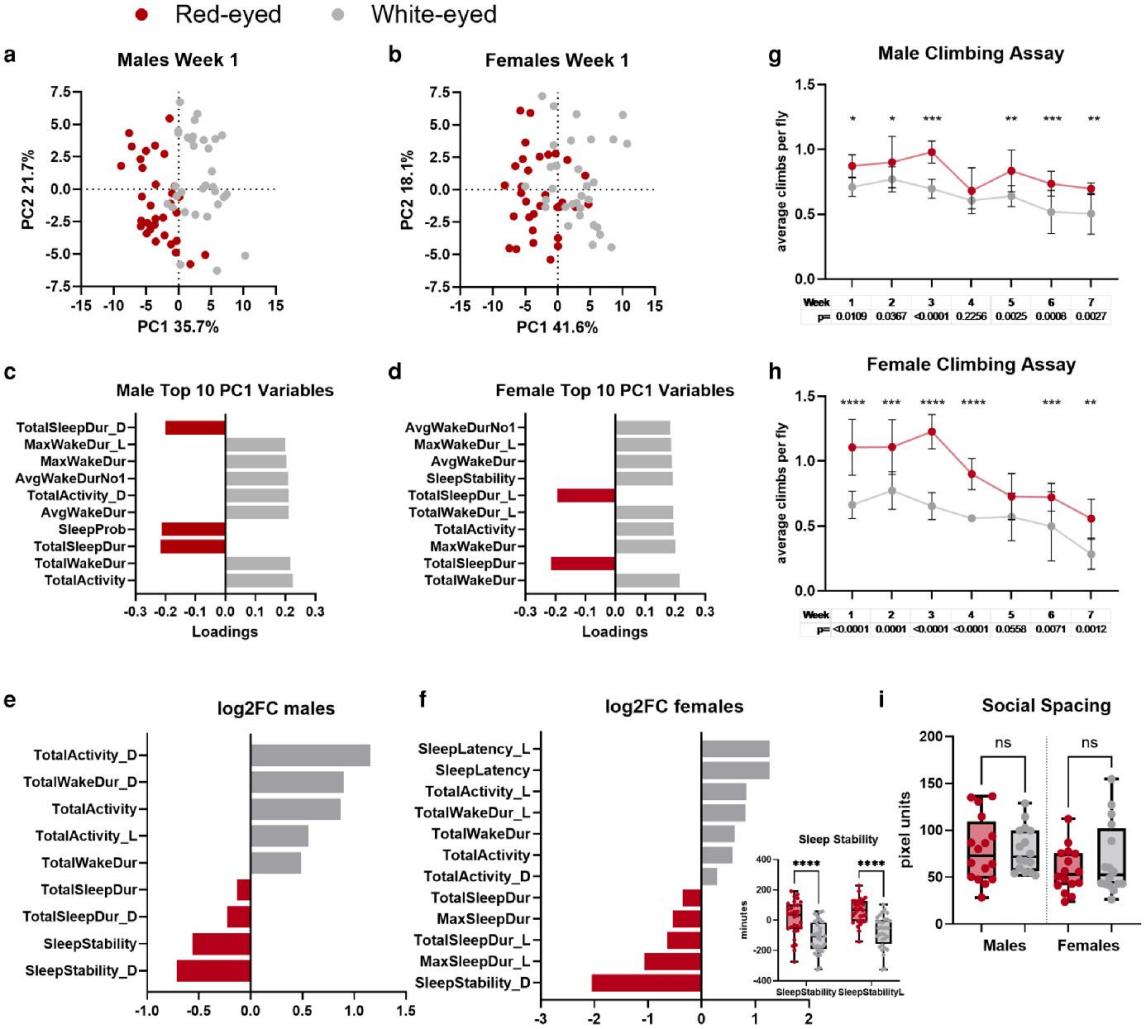
Behavioral phenotyping reveals differences in activity levels and age-related locomotor degeneration but not social spacing. For all panels, red-eyed flies are represented in a dark red hue and white-eyed flies are represented in a light gray hue. a, b) PCA of activity data. Each dot represents an individual fly, with its position reflecting the similarity in activity and sleep patterns as measured by SCAMP (Vecsey *et al*. 2024). Red- and white-eyed flies cluster separately along the first principal component (PC1), which explains 35.7% of the variance in males a) and 41.6% in females b). See also Supplementary Fig. 2. c, d) PCA loadings for the top 10 parameters output by SCAMP contributing to principal component 1 (PC1) for males and females, respectively. e, f) Log2 fold change for significant (*P* < 0.05) activity parameters in 1-week-old male and female flies, respectively. Due to the negative values, raw sleep stability is shown in the insert box plot ****P < 0.0001. For a)–f), *N* = 32 flies per group. g, h) Climbing ability of male and female flies, respectively. *P*-values for the multiple comparisons are shown below the graph. *N* = 5 replicates of 10 flies each for each climbing assay. The error bars represent 95% confidence intervals. i) Social spacing of males and females. Median distance to each individual's nearest neighbor was calculated. *N* = 16 replicates of 20 flies each.

Despite *white*'s role in visual processing and the biosynthesis of neurotransmitters, histamine, and melatonin (Borycz *et al*. 2008), a systematic analysis comparing wake/sleep patterns of w^+^ and w^−^ flies at different ages is currently lacking. *Drosophila* circadian rhythms are primarily driven by light, especially in controlled laboratory settings that minimize temperature fluctuations (Dubowy and Sehgal 2017). Aged wild-type and neurodegenerative disease model flies tend to experience changes in activity levels and disturbed sleep compared to younger or healthier counterparts (Koudounas *et al*. 2012; Chakravarti *et al*. 2017). We measured animal movement over time using the TriKinetics Drosophila Activity Monitor (DAM2) in conjunction with the analysis program SCAMP, which calculates 51 different activity and sleep behavior measures (Vecsey *et al*. 2024). w^−^ flies, while suffering from reduced visual acuity (Ferreiro *et al*. 2018), still exhibit functional circadian rhythms (Supplementary Fig. 2e–r). To capture how these variables might contribute to differences between w^+^ and w^−^ flies, we performed PCA of all measurements produced with SCAMP (Fig. 1a and b). The first principal component (PC1) separates w^+^ and w^−^ flies and accounts for 35.7 and 41.6% of variability in the male and female dataset, respectively. To determine the variables driving these differences, we isolated and plotted the top 10 measures contributing to PC1 (Fig. 1c and d). This analysis reveals in both sexes and across different ages that w^+^ flies show an increase in sleep-related parameters, while w^−^ flies show an increase in activity and wake parameters (Supplementary Fig. 2a–d). While the factors making up PC1 indicate changes in overall behavioral patterns, they cannot fully identify significant differences. For a more traditional statistical approach, we performed 2-way ANOVA using the SCAMP results. This analysis agrees with the PCA results, showing significantly increased sleep-related parameters for w^+^ flies and activity/wake-related parameters for w^−^ flies (Fig. 1e and f; Supplementary Table 1). In summary, we present the first account of activity and sleep behavior differences in backcrossed w^+^ and w^−^ flies. Our results show that while the circadian rhythm remains intact, w^−^ significantly affects sleep/wake patterns, leading to decreased sleep and increased activity levels.

Retinal degeneration is a common measure for neurodegeneration in *Drosophila*. Previous studies comparing w^1118^ to other wild-type strains, such as Oregon-R and Vallecas, have linked *white* mutation to progressive retinal degeneration, suggesting a potential role in age-related neurodegeneration (Ferreiro *et al*. 2018). However, this retinal degradation could simply be due to eye pigment loss and exacerbated over time by light exposure. To assess neurodegeneration independent of eye pigmentation, we measured locomotor ability using the RING assay (Anaka *et al*. 2008; Ambegaokar and Jackson 2010; Chakravarti *et al*. 2017). Previous studies reported poor locomotor ability in aged w^−^ flies, based on comparison between w^1118^ and wild-type strains such as Vallecas and Canton-S. However, these studies assessed limited time points (Qiu *et al*. 2017; Ferreiro *et al*. 2018). To evaluate the locomotor ability of our isogenic w^+^ and w^−^ flies throughout their approximate median lifespan, we tested them weekly from 1 to 7 weeks posteclosion. At all time points, w^−^ flies averaged worse locomotor function than w^+^ flies, with this difference being significant most weeks (Fig. 1g and h) in agreement with previous studies. However, the rate of locomotor deterioration remained similar between w^+^ and w^−^ flies as they aged. These findings suggest that *white* mutation impairs baseline neuromotor function but does not by itself accelerate age-related locomotor decline.

*Drosophila* social behavior is shaped by chemical cues such as scent and pheromones but can also be affected by visual cues (Aso *et al*. 2014; Rooke *et al*. 2020; Billeter *et al*. 2024). Previous studies comparing 4- to 5-day-old w^+^ Canton-S (CS) to w^−^ CS flies carrying the w^1118^ mutation (w^1118^Cs_10_) reported altered social spacing, potentially due to reduced visual acuity of *white* mutants (Simon *et al*. 2012). To determine whether our genetically matched w^+^ and w^−^ flies show similar effects, we performed the same social spacing assay and calculated nearest neighbor distances with the cohort. Unexpectedly, we detected no significant differences in social behavior between w^+^ and w^−^ flies (Fig. 1i). It is possible that the CS and w^1118^Cs_10_ lines used in the published study, while initially backcrossed, diverged over time due to genetic drift. Our results suggest that visual acuity differences are unlikely to drive social spacing behavior and emphasize the confounding role of genetic drift across laboratory stocks in behavioral assays.

### Metabolic phenotyping reveals sex-specific differences in triglyceride levels and starvation resistance

*White* is responsible for intracellular transport of small molecules, including metabolites such as tryptophan and guanine. Its mutation has been associated with changes in downstream metabolism in prior studies (Mackenzie *et al*. 2000; Badawy 2017; Hebbar *et al*. 2023). Altered tryptophan metabolism has also been linked to lipid metabolism in mice and human tissue culture and cholesterol homeostasis in flies (Myers *et al*. 2021; Luo *et al*. 2024). In rats, increased tryptophan levels have been associated with increased lipid peroxidation, a common readout for oxidative stress (Aviram *et al*. 1991). While we did not directly measure tryptophan metabolites in this study, we selected metabolic assays based on *white's* established role in metabolism. To control for developmental and age-related variation, all metabolic assays were performed on 8-day-old adult flies under standard 12-h light:dark cycles.

**Fig. 2.**
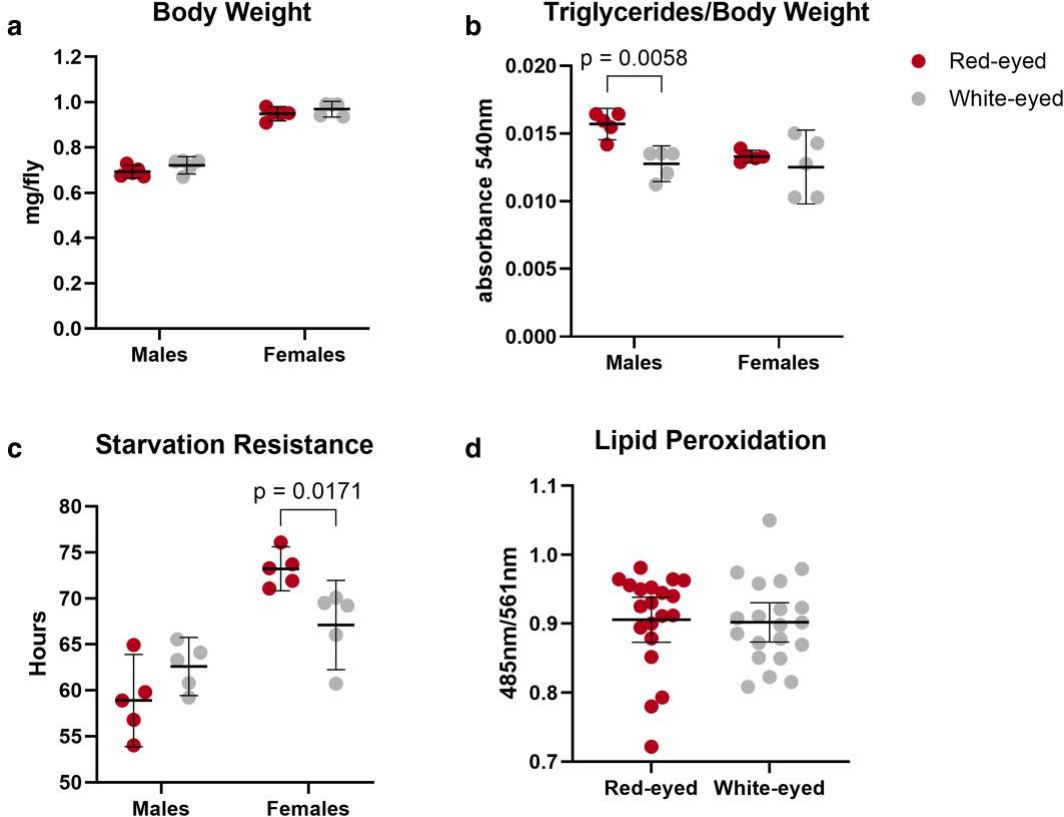
Mutation of the *white* gene affects triglyceride levels and starvation resistance in a sex-specific manner. a) Body weight per fly of w^+^ and w^−^ males and females. b) Triglyceride content per body weight of fly compared for males and females. For a) and b), *N* = 5 replicates of 10 pooled flies. c) Starvation resistance measured by the number of hours for all flies in a replicate to die. *N* = 5 replicates of 20 flies each. d) Lipid peroxidation measured by absorbance ratio using BODIPY 581/591 C11 (Invitrogen). *N* = 20 replicates of 10 individuals each. All error bars represent the mean with 95% confidence interval.

We first measured body weight and triglyceride content. No difference in body weight was observed between w^+^ and w^−^ flies in either sex (Fig. 2a). However, w^−^ males show significantly lower triglyceride content than w^+^ males (Fig. 2b), with no significant differences observed in females.

Because triglyceride content can influence starvation resistance, we next performed a starvation resistance assay (Oudman *et al*. 1994). A previous study comparing w^1118^ with Oregon-R, and Vallecas wild-type strains reported reduced starvation resistance w^−^ flies of both sexes, although triglyceride content was not assessed (Ferreiro *et al*. 2018). In our backcrossed lines, we found that w^−^ females had significantly reduced starvation resistance compared to w^+^ females, while no difference was observed in males (Fig. 2c). These results partially validate previous findings (Ferreiro *et al*. 2018) and, when considered alongside our triglyceride measurements, suggest that *white* mutation affects metabolic phenotypes in a sex-specific manner. Notably, triglyceride levels and starvation resistance did not show a consistent relationship across sexes, indicating that additional factors beyond energy stores likely contribute to the observed survival differences.

Oxidative stress is linked to neurodegeneration in both humans and flies, and w^1118^ flies have lower resistance to oxidative stress from paraquat or H_2_O_2_ exposure compared to Oregon-R and Vallecas (Ferreiro *et al*. 2018). These findings suggest a link between *w* mutation and oxidative stress. To test whether *white* affects oxidative stress, we measured lipid peroxidation levels in embryos. As w^+^ and w^−^ embryos are indistinguishable, we generated homozygous w^+^ and w^−^ embryos from our isogenic crosses for these experiments. No significant differences were observed (Fig. 2d), suggesting that under baseline conditions, *white* mutation does not alter oxidative stress sensitivity in early development.

### Fitness phenotyping reveals a difference in mated egg laying

**Fig. 3.**
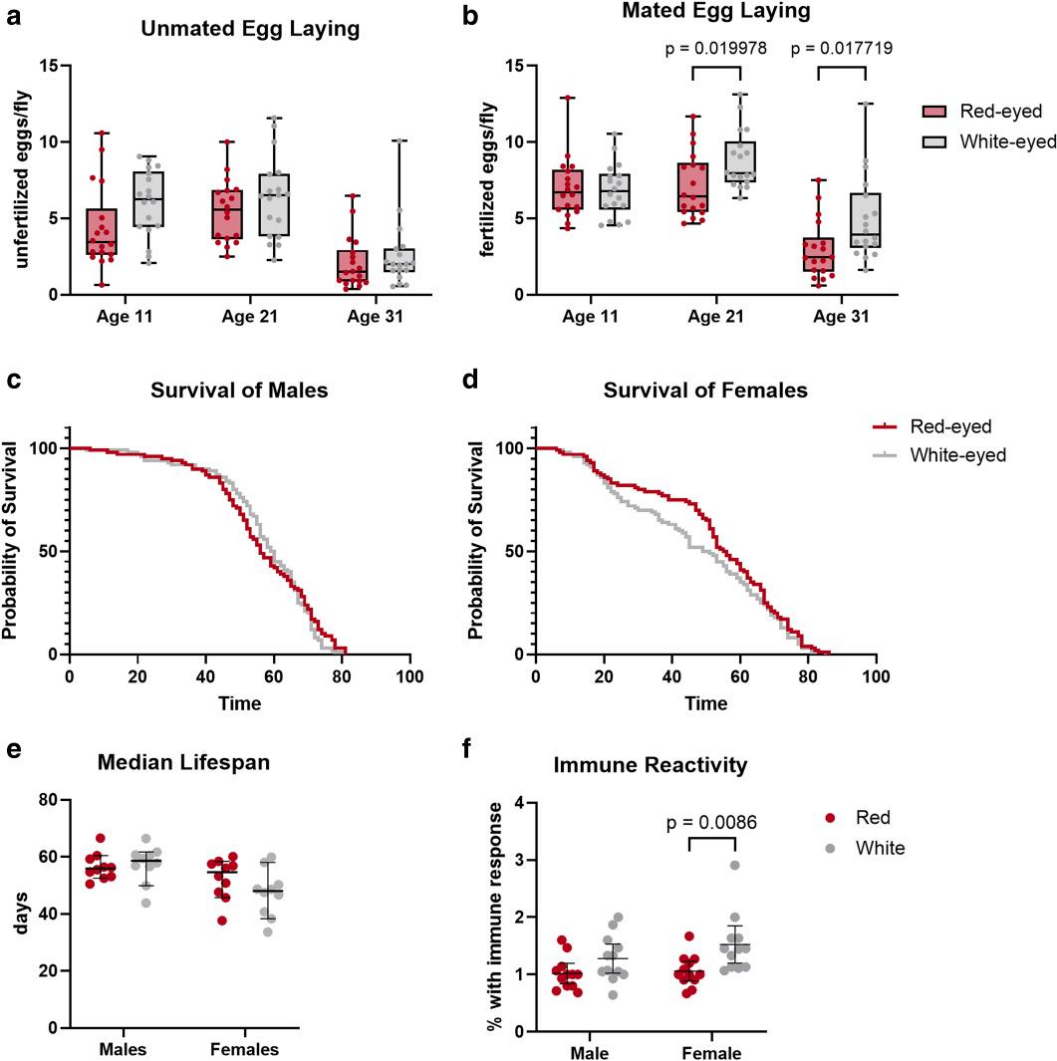
The *white* gene affects mated egg laying. a, b) Eggs laid per unmated and mated females respectively at 11, 21, and 31 days of age. *N* = 6 replicates of 20 females over 3 days. c, d) Survival curve for males and females, respectively. e) The median lifespan of red- and white-eyed males and females shows no significant difference between w^+^ and w^−^ males and females. *N* = 10 vials of 10 flies each. Error bars represent the median with 95% confidence intervak. f) Immune reactivity measured as the % of larvae with immune response normalized to the median red eye value. *N* = 9 replicates of 20 larvae each. Error bars represent the mean with 95% confidence interval.

Egg production requires both energy and nutrients, with protein availability being particularly important (Mirth *et al.* 2019). Despite the role of *white* in tryptophan transport, its effects on egg laying have not been studied. To assess egg laying behavior, we counted the number of eggs laid by unmated and mated females at 11, 21, and 31 days of age. We observed no significant difference in egg production by unmated females; however, mated w^−^ females laid significantly more eggs than w^+^ females at both 21 and 31 days (Fig. 3a and b), despite previous reports of reduced heterosexual copulation success in w^−^ males (Hing and Carlson, 1996; Krstic *et al.* 2013; Xiao *et al.* 2017). To determine whether this increase reflected differences in fertility or embryo viability, we conducted additional hatching and embryo survival assays. We observed no significant differences in average time to hatch, number of eggs hatched per hour, or total embryo survival between homozygous w^+^ and w^−^ lines (Supplementary Fig. 3). These results suggest that *white* mutation affects mated egg laying behavior but not fertilization success or embryonic viability.

Previous studies using w^1118^, Canton-S, and Oregon-R have reported differences in lifespan between w^+^ and w^−^ lines (Oxenkrug 2010; Qiu *et al*. 2017; Ferreiro *et al*. 2018). Notably, these studies reported conflicting results. For example, w^−^ w^1118^ flies have been shown to live longer than Oregon-R, but no differences were observed between w^1118^ and Canton-S flies, while Oregon-R flies were found to live significantly longer than Canton-S flies (Ganetzky and Flanagan 1978; Oxenkrug 2010; Qiu *et al*. 2017). These discrepancies point toward genetic background as a contributing factor. To clarify the role of *white*, we measured lifespan in our backcrossed w^+^ and w^−^ lines. We observed no significant difference in median lifespan or survival curves between genotypes in either sex (Fig. 3c–e), indicating that the previously reported changes were due to genetic background variation rather than the *white* mutation.

In humans, extensive research indicates a link between tryptophan metabolism, inflammation, and the immune system (Seo and Kwon 2023). A similar link has been proposed for *Drosophila*, where metabolites of the kynurenine pathway were shown to interact with zinc and influence the immune system (Garay *et al*. 2022). However, the effect of *white* mutation on immune function has not been previously tested. Based on this rationale, we assessed innate immune reactivity in third instar larvae from w^+^ and w^−^ lines. We observed a sex-specific difference: w^−^ females exhibited an increase in immune reactivity, while no difference was observed in males (Fig. 3f). These results suggest that *white* may influence immune signaling in a sex-dependent manner.

### RNA-seq reveals general and sex-specific gene expression changes

Given the potential far-reaching effects of *white* mutation on metabolism and the observed differences in behavior, we performed gene expression analysis on heads from 1-week-old w^+^ and w^−^ male and female flies to assess the system-wide effects of *white mutation*.

**Fig. 4.**
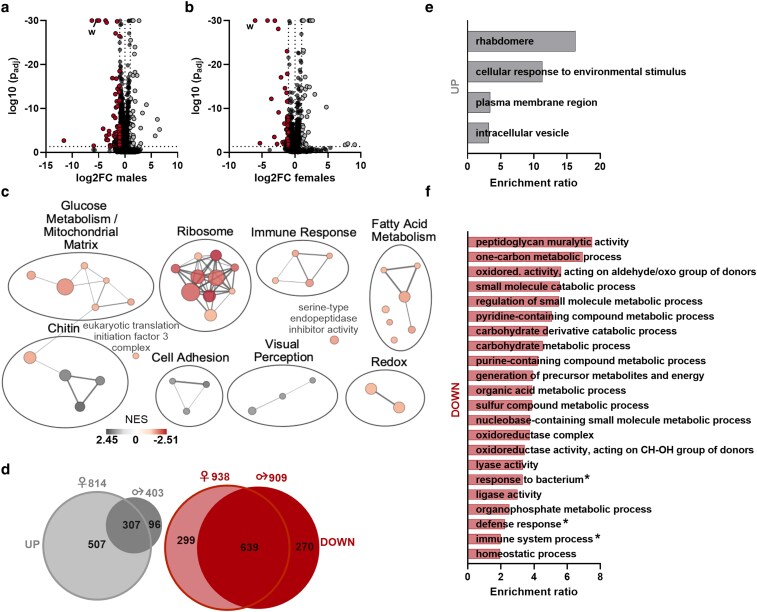
RNA-seq reveals significant both broad and sex-specific gene expression changes. a, b) Volcano plots of DE genes for males a) and females b). Expression of *w* is significantly lower in both male and female w^−^ flies. See also Supplementary Fig. 3a. c) GSEA. Plotted are only significant pathways (*Q* = 0.1) and edges with 0.1 similarity. See also Supplementary Tables 2 and 3 and Supplementary Fig. 3b. d) Significantly (*P* < 0.05) differentially upregulated (left/gray diagram) and downregulated (right/red diagram) genes in females (left circle) and males (right circle). The number above each circle is the total number of up or downregulated genes for that condition, with the numbers inside the Venn diagram showing how many of those genes are unique or shared between males and females. See also Supplementary Tables 6–9. e, f) Overrepresentation analysis for up- and downregulated male-specific DE genes (*P* < 0.05) respectively. See also Supplementary Fig. 4c and d and Supplementary Tables 10–13.

Differential gene expression analysis revealed 1,312 and 1,752 total DE transcripts in males and females, respectively, with more genes downregulated than upregulated in w^−^ flies of both sexes. As expected, we detected a >99% decrease in *white* expression in w^−^ flies in both males and females (Fig. 4a and b; Supplementary 4a). To better interpret the other gene expression changes, we performed GSEA. While females had a greater number of enriched pathways, the overall results for males and females were similar, with an overlap of many gene sets (Fig. 4c; Supplementary Fig. 4b and Supplementary Tables 2–5). Consistent with the effects of *white* on metabolism, our analysis reveals related pathways, such as glucose and fatty acid metabolism as significantly altered in both sexes.

The sex-specific effects seen in our phenotyping analysis prompted us to investigate sex-specific transcriptional changes across the genome. Despite a significant overlap in DE genes between sexes, we identified 366 and 806 DE genes unique to males (Supplementary Tables 6 and 7) or females (Supplementary Tables 8 and 9), respectively (Fig. 4d). We performed overrepresentation analysis on these sex-specific DE genes to identify related transcriptional programs. Male-specific upregulated genes were mostly related to response to environmental stimuli, while the male-specific downregulated genes mainly involved carbohydrate and nucleotide metabolism (Fig. 4e and f; Supplementary Tables 10 and 11). Interestingly, w^−^ males show a depletion of genes involved in immune response pathways, with antimicrobial peptides such as AttB/C/D, CecA1/2, DptB, and Mtk among the leading-edge genes, suggesting reduced immune activity or potential suppression of immune signaling. This observation reinforces our findings from the immune assay and suggests that differences in immune response are not limited to the larval stage. The top 10 female-specific upregulated genes were enriched for development-related pathways, while the female-specific downregulated genes involved ribosomes, small noncoding RNAs, and small molecule metabolism (Supplementary Fig. 4c and d; Supplementary Tables 12 and 13). In summary, our transcriptomic analysis reveals broad differences in gene expression between w^−^ and w^+^ flies, as well as additional sex-specific effects, further supporting our phenotypic observations.

## Discussion

*White* widely used as phenotypic marker in *Drosophila*, under the assumption that its mutations have minimal side effects. Using flies backcrossed for 10 generations, we show that w^−^ and w^+^ flies show differences in metabolic, immune, and behavioral phenotypes. While our results replicate some previously reported findings, they also diverge from studies that used different genetic backgrounds, highlighting genetic drift and background as major confounding factors. We also present a rigorously controlled transcriptomic dataset, which reveals widespread gene expression differences between w^+^ and w^−^ flies, with functional enrichment in metabolic, immune, and developmental pathways.

Advances in science and improved methods reveal increasingly subtle traits and may uncover previously hidden effects of mutations long used as convenient markers. Given the widespread use of *Drosophila* in disease research, clarifying the effects of w^−^ is critical for accurate interpretation. Here, we employed widely used, sensitive, state-of-the-art phenotyping assays to establish a baseline behavioral and physiological assessment of w^−^. These results will help researchers interpret their findings and make informed decisions when selecting or modifying genetic backgrounds.

Our results highlight genetic background as a major driver of phenotypic differences. It is important to note that this study used a single w^+^ donor allele backcrossed into a single w^−^ line. Given the prominent background effects we identified, and the absence of genetic rescue experiments, it remains possible that a different w^−^ background or alternative white allele would yield different results. Furthermore, our design does not fully account for linkage disequilibrium. That said, we do not observe an enrichment of DE genes on the X chromosome relative to the autosomes, despite the reduced opportunity for recombination due to the absence of male meiotic crossover. However, regions of particularly low recombination, such as telomeres and centromeres, may still harbor linked variants that are not fully equilibrated between lines. Future studies using defined genomic manipulations and alternative white alleles will be critical to fully disentangle the role of *white* from neighboring genomic variation.

The *Drosophila* community commonly compares phenotypes in lines with different genetic backgrounds; our results underline the importance of including proper controls such as deficiency strains, multiple independently derived alleles of the same gene, rescue experiments, phenotypic comparison of F1 hybrids, and backcrossing into isogenic backgrounds. Efforts should also be made to reduce genetic drift within lab stocks, including increased strain tracking, avoidance of extensive bottlenecking during husbandry and regular exchange of individuals between duplicate stock vials.

Similar effects could also exist for other popular genetic markers. For example, intracellular transport proteins scarlet and brown heterodimerize with white, and their mutations likely share some phenotypic and molecular effects with *w* mutants (Ferreiro *et al*. 2018). The curly wing phenotype is caused by mutation of *duox*, a reactive oxygen species generating NADPH oxidase that plays a role in the immune system (Hurd *et al*. 2015). Stubble, a transmembrane serine protease, plays an important role in shaping the cytoskeleton, and homozygous mutations are lethal in larvae, indicating its crucial role in *Drosophila* biology (Appel *et al*. 1993).

The issue of genetic drift is not unique to *Drosophila*. The mouse line C57BL/6 (commonly called black 6) has many substrains, each with phenotypic differences due to hundreds of generations isolated in various labs and repositories. The Jackson Laboratory, originator of black 6, considers 20 generations of separation sufficient to create a substrain (Bryant 2011; Jackson Lab 2013; Mekada and Yoshiki 2021). Assuming a generational time of 14 days (i.e. flipping every 2 weeks), *Drosophila* produces 26 generations per year. According to this estimate, the *white* mutant isolated by Morgan in 1910 (w^1^) has spent nearly 3,000 generations as laboratory stock. Over the years, it has also been distributed to countless individual labs and stock centers. As a result, the potential for genotypic and phenotypic differences between different stocks of the same original fly line is substantial, which helps explain phenotypic discrepancies reported in the literature.

## Supplementary Material

iyaf097_Supplementary_Data

## Data Availability

Requests for further information and resources should be directed to and will be fulfilled by the lead contact, Adelheid Lempradl (Heidi.Lempradl@vai.org). Strains are available from the corresponding author upon request. The raw RNA-sequencing (RNA-seq) data are available on Gene Expression Omnibus under the accession code GSE290227. The code for RNA-seq analysis is available in the supplemental files. Any additional information to reanalyze the reported data is available from the lead contact upon request. Supplemental material available at GENETICS online.
