# Supplementary material for: More than meets the eye: mutation of the white gene in Drosophila has broad phenotypic and transcriptomic effects
Source: Genetics. 2025 May 17;230(3):iyaf097. doi: 10.1093/genetics/iyaf097 (PMC12239204; doi:10.1093/genetics/iyaf097)
Supplement: iyaf097_Supplementary_Data [file iyaf097_supplementary_data.zip › Supplemental_Figure_Legends_GENETICS-2025-308138.docx]

**Figure S1:** **Backcrossing schematic**

Cross 1 was between a Harwich 15 female and a w^1118^ male. Virgin female offspring were collected and mated to w^1118^ males for cross 2. Red-eyed female virgins from the previous cross were mated w^1118^ males for crosses 2-10. Flies were collected for assays from cross 10. Flies from cross 10 were also crossed to one another to produce homozygous white-eyed and red-eyed lines for experiments requiring embryos and larvae, which cannot be phenotyped by eye color. Created in BioRender. Rickle, A. (2025) https://BioRender.com/g63b773

**Figure S2:** **Additional activity assay data shows decreased activity and increased sleep over time in both sexes, related to figure 1**

**a and b)** Total activity counts for males and females respectively over 7 weeks. **c and d)** Total sleep duration for males and females respectively over 7 weeks. **a-d)** Error bars represent the mean with 95% CI. N = 32 flies per group. **e-r)** Average total activity counts shown in half-hour bins for 1 week old (e, f), 2 week old (g, h), 3 week old (i, j), 4 week old (k, l), 5 week old (m, n), 6 week old (o, p), and 7 week old (q, r) males (left column) and females (right column). N = 32 flies per group.

**Figure S3: There is no significant difference in hatch rate or embryo survival, related to figure 3**

For all panels N=3 replicates of 100 embryos each, α=0.05. **a)** Average time to hatch. Error bars represent a 95% confidence interval. **b)** Number of embryos counted as hatched each hour. Bars represent the average number with a 95% confidence interval. F=0.05252 **c)** Percent embryo survival.

**Figure S4:** **RNA-seq confirms loss of *w* expression, female-specific gene expression changes, related to figure 4**

**a)** Expression of *w* in male and female w^+^ and w^-^ flies. Error bars represent the mean with standard deviation. **b)** Gene Set Enrichment Analysis (GSEA) for female flies. Plotted are only significant pathways (Q=0.1) and edges with 0.1 similarity. See also tables 4 and 5. **c and d)** Overrepresentation analysis for up- and down-regulated female-specific DE genes (p<0.05) respectively. See also tables 12 and 13.
